# Elevated expression of Toll-like receptor 7 and its correlation with clinical features in patients with primary Sjögren’s syndrome

**DOI:** 10.1186/s42358-024-00360-4

**Published:** 2024-03-04

**Authors:** Huimin Yang, Chao Sun, Xin Wang, Tao Wang, Changhao Xie, Zhijun Li

**Affiliations:** 1 Jinan University, Guangzhou, China; 2 Department of Rheumatology and Immunology, Second Affiliated Hospital of Bengbu Medical College, Anhui, China; 3 Key Laboratory of Infection and Immunity of Anhui Higher Education Institutes, Bengbu Medical College, Anhui, China; 4 Department of Rheumatology and Immunology, The First Affiliated Hospital of Bengbu Medical College, No. 287 Changhuai Road, 233004 Bengbu, Anhui China

**Keywords:** Autoantibody, Clinical features, Immunoglobulin, Toll-like receptor 7, Sjögren's syndrome

## Abstract

**Background:**

The labial salivary glands (LSGs) are important for the diagnosis, evaluation of therapeutic efficacy, and genetic analyses of primary Sjögren’s syndrome (pSS). In autoimmune diseases, the recognition of self nucleic acids and viral RNA and DNA through endogenous Toll-like receptor(TLR) triggers the production of type I IFN and pro-inflammatory cytokines, leading to the occurrence and progression of the disease. Here, we detected the expression of TLR7 in LSGs and analyse its correlation with clinical features and serum cytokines in pSS patients.

**Methods:**

LSGs and serum samples were obtained from 56 pSS patients and 19 non-SS patients (non-pSS patients). The expression of TLR7 in the LSGs was evaluated with immunohistochemistry. The serum levels of interferon-α (IFN-α) and interleukin-6 (IL-6) were quantified by ELISA. Laboratory parameters were measured by clinical standard laboratory techniques.

**Results:**

TLR7-positive cells in pSS were localized in the ductal epithelial cells and lymphocytes of LSGs. The expression of TLR7 was upregulated in pSS patients compared with controls. Patients with anti-Ro52 antibody positivity showed higher TLR7 levels than those who were negative but not those with anti-Ro60 and anti-SSB. TLR7 levels were positively associated with the levels of IgG, IgA, ANA, IL-6, IFN-α and serum globulin but were not associated with IgM, C3, C4, or rheumatoid factor (RF) in serum.

**Conclusion:**

TLR7 may be involved in the inflammatory response and the production of antibodies in pSS and plays an important role in local and systemic pSS manifestations. This research showed that TLR7 is involved in pSS pathogenesis.

## Introduction

Primary Sjögren’s syndrome (pSS) is a chronic systemic autoimmune disorder characterized by the infiltration and destruction of exocrine glands, primarily salivary and lacrimal glands, leading to keratoconjunctivitis sicca and xerostomia [1]. pSS is commonly characterized by lymphocytic mononuclear infiltration in the affected target organs and hypergammaglobulinemia and autoantibody production by abnormal activation of B lymphocytes [2].

At present, the aetiology and pathogenesis of Sjögren’s syndrome are still unclear. Infection has long been considered a possible major environmental risk factor for pSS. Specifically, there is increased activity in key parts of the innate immune system that defend against viruses, including Toll-like receptor (TLR) signalling and the type I interferon (IFN) system. Toll-like receptors (TLRs) are one of the cellular transmembrane receptors and pattern recognition receptors (PRRs) of the intrinsic immune system discovered recently. TLRs can transmit signals to the cell to induce a cellular immune response and act as a bridge between innate immunity and adaptive immunity. They can recognize bacteria, mycoplasma, viruses and other pathogenic microorganisms; activate the innate immune response; and produce costimulatory molecules and a large number of proinflammatory factors to induce adaptive immunity [3, 4]. TLR7 is an important member of the TLR family, which is mainly related to virus recognition. They participate in the antiviral response by recognizing single-stranded RNA (ssRNA) viruses. A number of experiments have demonstrated that TLR7 signalling plays an important role in the pathogenesis of autoimmune diseases, inducing the release of proinflammatory cytokines such as IL-6 and IFN-α, leading to the activation of B cells [5–7].

Increased expression of TLR7 in lupus-prone mice as a key upstream driver is associated with disease etiopathology. TLR7 signals induce increased type I IFN gene transcripts, the activation of self-reactive B cells and the release of inflammatory cytokines such as IL-6, IL-10 and TNF, resulting in autoimmune disease manifestations. In lupus-prone mice, blocking TLR7 improves or prevents the onset of lupus [8].

TLR7 signalling has been demonstrated to play an important role in the occurrence and development of SLE [9]. However, its role in pSS is poorly understood. SLE and SS are two closely related autoimmune diseases. The two diseases share many common features, including epidemiological features, clinical manifestations, serological features and genetic risk factors, and researchers have proposed that SLE and SS may have similar associations in their pathogenesis [10]. In addition, IFN-α is the most important subtype of type I IFN. Multiple omic studies have shown that there is a correlation between serum IFNα concentrations and type I IFN characteristics [11]. Circulating IFN-α was significantly associated with autoantibodies and B-cell activation markers such as BAFF in patients with pSS [12].

Although previous studies showed increased TLR7 expression in pSS labial glandular tissue, the small sample size and the correlation between TLR7 expression and serum cytokines and clinical features of pSS patients were not analysed. Therefore, the purpose of this study was to determine the expression of TLR7 in the labial glandular tissue of patients with pSS, to measure cytokines in peripheral blood and to analyse the correlation between TLR7 and clinical features and related cytokines in patients with pSS.

## Methods

### Patients

Fifty-six patients with pSS and 19 control subjects were enrolled in this study in the Department of Rheumatology and Immunology, the Second Affiliated Hospital of Bengbu Medical College, Bengbu, between 2021 and 2022. The pSS diagnosis was made according to the 2016 American College of Rheumatology/European League Against Rheumatism classification criteria [13]. The control group included individuals complaining of arthrodynia, sicca symptoms or positive antinuclear antibodies who did not fulfil the SS criteria. The clinical characteristics of the pSS patients and non-SS patients are summarized in Table 1. Anticoagulant containing heparin sodium was used to store the blood samples. Sera were collected and kept in storage at at -80 °C pending additional analysis. The lip gland tissue was fixed with 10% formaldehyde solution and embedded in paraffin, and the section thickness was 4 μm.

This study was approved by the Ethics Committee of the Second Affiliated Hospital of Bengbu Medical College (approval no. 2023BYEFY-5 A).

### Histology and immunohistochemical staining

LSG samples from 56 pSS patients and 19 non-SS patients were fixed in formalin, embedded in paraffin, and sectioned. These sections were stained with haematoxylin and eosin (H&E) and examined by an independent pathologist. The focus score (FS) is the total number of foci per 4 mm^2^ salivary gland tissue [13, 14]. A focus is defined as an aggregate of ≥ 50 lymphocytes. Immunohistochemical staining for TLR7 in each specimen was performed. Tissue slides were probed with primary antibodies at the indicated concentrations overnight at 4 °C, followed by incubation with HRP-conjugated secondary antibody at room temperature for 1 h. The polyclonal antibodies of rabbit antihuman TLR7 (working dilution 1:150; 17232-1-AP, Proteintech) were used in this experiment; the results were stained with 3,3’-diaminobenzidine (DAB). Images were taken using a digital microscope colour camera (Leica Microsystems, Tokyo, Japan).

### Immunohistochemical analysis

The slide was scanned by a panoramic scan (3D Histech) and was analysed by ImageJ (downloaded by https://imagej.net/software/fiji/downloads), which was used to measure the TLR7 present in the tissue. First, digital images needed to be colour deconvoluted into three layers: DAB, haematoxylin, and a complementary image. Only the DAB colour layer representing staining positivity was analysed considering the pixel intensity values in the range of 0–255 (wherein 0 represents the darkest shade of the colour, and 255 represents the lightest shade of the colour as standard) as previously described [15]. The mean intensity default threshold was set at the ‘Image’ menu, and using the ‘Measure’ tool from the ‘Analyse’ menu, the percentage of positive pixels within the selected area was determined.

### ELISA

Plasma levels of cytokines were quantified using enzyme-linked immunosorbent assays (ELISA) in the validation cohort, according to the manufacturer’s instructions. The following ELISA kits were used: IL-6 (DL-IL6-Hu, Dldevelop, Wuxi, China) and IFN-α (DL-IFNα-Hu, Dldevelop, Wuxi, China).

### Statistical analysis

Statistical analysis was performed using SPSS Statistics 26 and GraphPad Prism 6 software. Data were analysed using the Shapiro–Wilk normality test, the Kruskal–Wallis test, and Mann–Whitney’s U test with post hoc Dunn’s multiple comparison test. In addition, Spearman’s rank correlation test in nonparametric data was performed to determine the associations between TLR7 and clinical parameters and cytokines. Differences were considered statistically significant when *P-value < 0.05*.

## Results

### Clinical characteristics of pSS patients

The clinical characteristics of these pSS patients and non-SS patients are summarized in Table 1. It summarizes the characteristics of 56 patients and 19 age-matched controls. A total of 96.4% of pSS patients were female. The levels of anti-Ro/52 antibody, anti-Ro/60 antibody and ANA, serum globulin, RF, serum IgG and IgA in the pSS group were significantly higher than those in the control group, while the levels of C4 were significantly lower than those in the control group.

**Table 1:** Clinical features of pSS patients and controls

Variables	pSS (N = 56)	Non-SS (N = 19)	P value
Sex, female/male	54/2	17/2	0.244
Age, y	49.3 ± 14.0	53.1 ± 11.9	0.292
Disease duration, mo, M(P_25_ ~ P_75_)	33(26–41)	13(9–16)	0.080
Anti-Ro/52 antibody, n(%)	43(76.8)	2(10.5)	< 0.001
Anti-Ro/60 antibody, n(%)	31(55.4)	3(15.8)	0.003
Anti-La/SSB antibody, n(%)	19(33.9)	1(5.3)	0.015
RF,O-20IU/mL	49.1 ± 49.14	11.63 ± 12.62	< 0.001
ANA ≥ 1:80, n(%)	47(83.9)	3(15.8)	< 0.001
lgG,7–16 g/L	17.37 ± 6.51	11.95 ± 2.69	0.001
lgA,0.4–2.8 g/L	3.03 ± 1.83	2.42 ± 4.51	0.001
lgM,0.7-3.0 g/L	1.73 ± 1.81	1.09 ± 0.76	0.128
C3,0.88–2.01 g/L	0.94 ± 0.24	1.01 ± 0.23	0.281
C4,0.16–0.47 g/L	0.22 ± 0.10	0.28 ± 0.06	0.010
Serum Globulin,15–35 g/L	34.75 ± 9.03	27.01 ± 4.29	0.001
ESR, 0–20 mm/h	29.79 ± 27.59	11.47 ± 6.82	< 0.001
CRP, 0–6 mg/L	7.18 ± 10.76	5.34 ± 5.55	0.342
Schirmer ≤ 5 mm/5 min (%)	30(53.6)	12(63.2)	0.555
ESSDAI score	8.88 ± 6.19	NA	-
ESSPRI score	3.29 ± 1.10	NA	-

pSS, primary Sjögren’s syndrome; Non-SS, non-pSS patients; RF, rheumatoid factor; ANA, antinuclear antibodies; IgG, immunoglobulin G; IgA, immunoglobulin A; IgM, immunoglobulin M; C3, complement 3; C4, complement 4; CRP, C-reactive protein; ESR, erythrocyte sedimentation rate; ESSDAI, Disease activity of pSS patients was determined by the EULAR Sjögren’s syndrome disease activity index; ESSPRI, EULAR Sjögren’s Syndrome Patient Reported Index.

### The expression of TLR7 was significantly increased in the LSGs of pSS patients

**Fig. 1:**
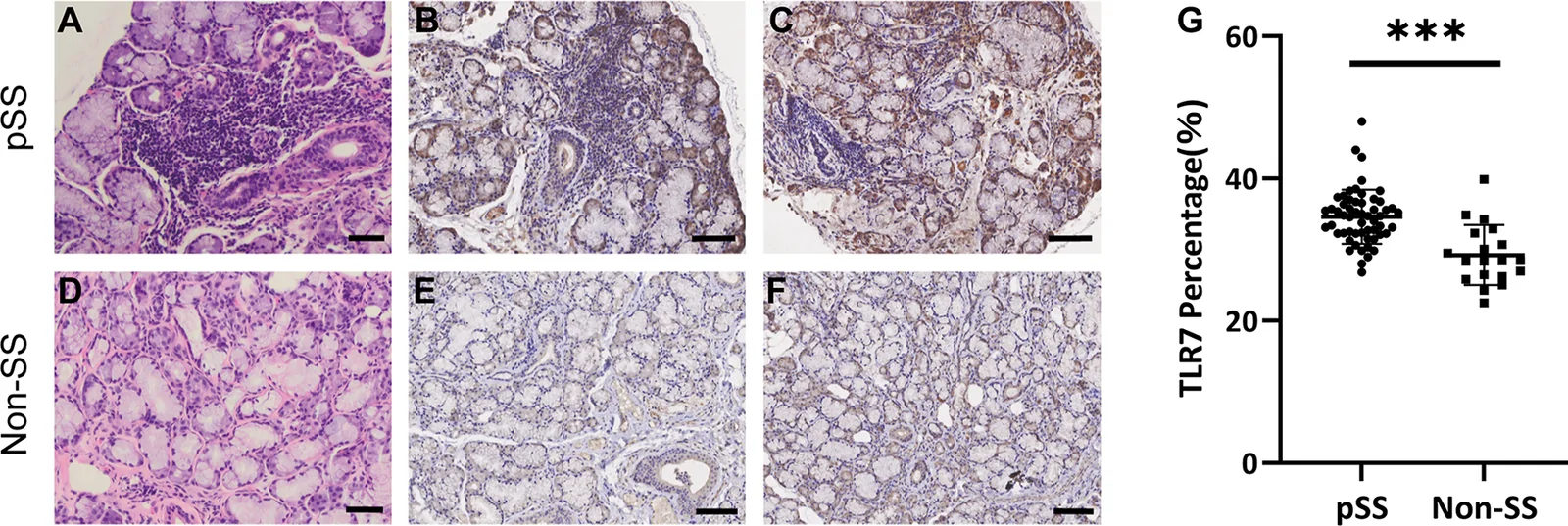
Expression of TLR7 in LSGs from pSS patients and non-SS control subjects. **A** and **D** H&E staining in LSGs. **B** and **C** High expression of TLR7 in pSS patients. **E** and **F** The lower expression of TLR7 in non-SS patients. Total magnification ×100 (bar indicates approximately 100 μm). **G** Significant differences in the percentage of staining positivity were observed between pSS patients and non-SS sicca control subjects (34.61 ± 3.79%, 29.24 ± 4.22%, *p* < 0.001). ***P < 0.001. pSS, primary Sjögren’s syndrome; Non-SS, non-pSS patients. TLR7, Toll-like receptor 7

LSG biopsies were analyzed according to hematoxylin and eosin (H&E) stained. It showed a higher lymphocytic infiltration and inflammation compared with the controls(Fig. 1A and D). TLR7 expression displayed a homogeneous distribution among mononuclear cells (MNCs). The expression levels of TLR7 were stronger in infiltrating MNCs and ducts of LSGs from the pSS patients than in those from the control subjects (Fig. 1B, C, E and F). ImageJ analysis revealed differences in the percentage of TLR7-positive cells in pSS patients (pSS = 34.61 ± 3.79% vs. non-SS = 29.24 ± 4.21%, *p* < 0.001; Fig. 1G).

### The levels of IFN-a and IL-6 were significantly increased in the serum of pSS patients; in addition, TLR7 expression in LSGs was correlated with the levels of IFN-a and IL-6 in peripheral blood

**Fig. 2:**
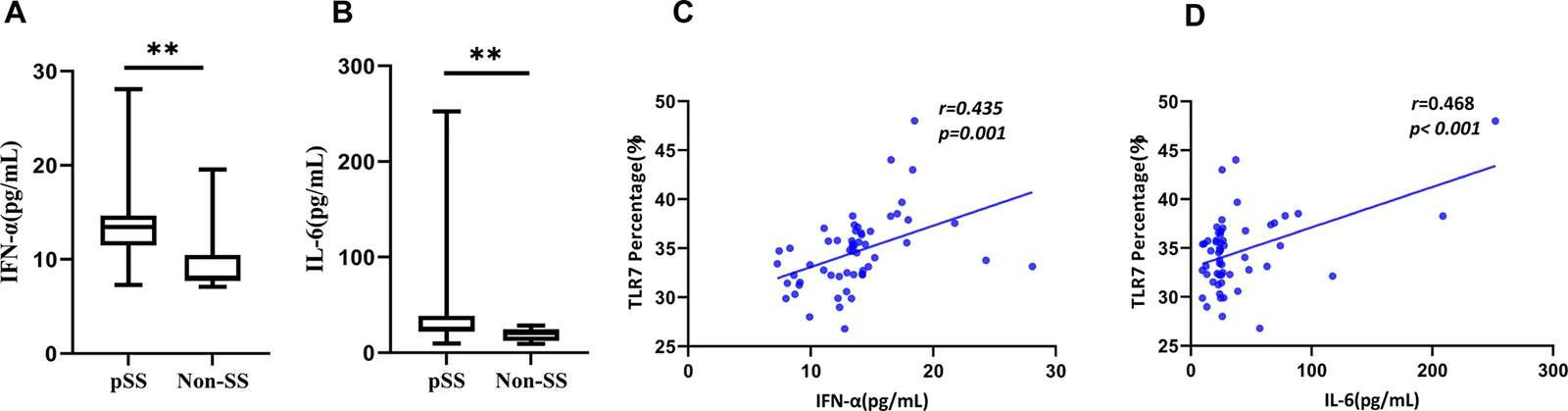
Serum levels of IFN-a and IL-6 in patients with pSS were elevated compared with those in non-SS patients. Correlations between TLR7 expression and serum cytokines. **A** TLR7 expression was positively associated with IFN-α. **B** TLR7 expression was positively associated with IL-6. **C** TLR7 expression in LSGs were positively correlated with serum IFN-a in pSS. **D** TLR7 expression in LSGs was positively correlated with the serum level of IL-6 in pSS. **P < 0.01. IFN-α, interferon-α; IL-6, interleukin-6

As shown in Fig. 2, the levels of IFN-a and IL-6 in pSS patients were 13.60 ± 3.91 pg/mL and 39.23 ± 42.99 pg/mL, respectively, while in non-SS patients, the levels were 10.37 ± 4.20 pg/mL and 18.83 ± 6.37 pg/mL, respectively (Fig. 2A and B). The levels of IL-6 and IFN-a in pSS patients were significantly higher than those in non-SS patients (*p* < 0.01). TLR7 expression in LSGs was correlated with the levels of IFN-a in peripheral blood (*r* = 0.435, *p* = 0.001; Fig. 2C) and IL-6 (*r* = 0.395, *p* < 0.001; Fig. 2D) in pSS.

### The expression of TLR7 in LSGs was associated with autoantibody production in pSS patients

**Fig. 3:**
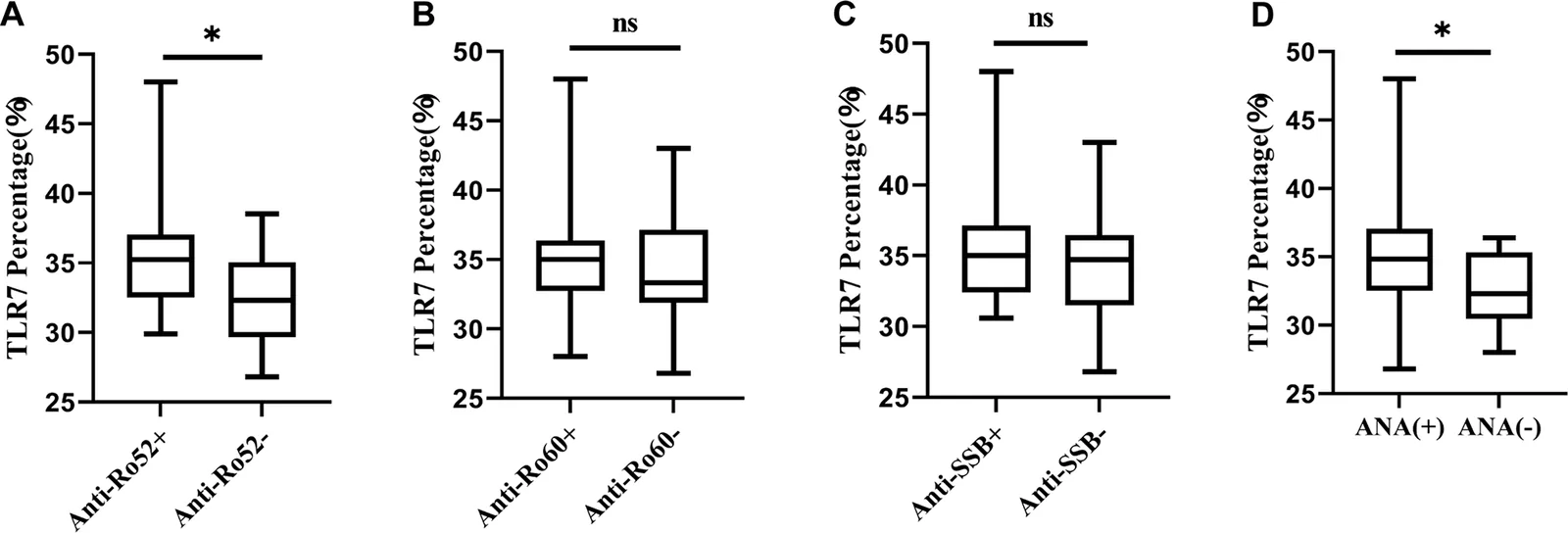
The correlations between TLR7 levels and autoantibodies in pSS. **A** Patients with anti-Ro/52 antibody positivity had higher expression of TLR7 than those who were negative. **B** and **C** There were no statistically significant differences between the anti-Ro/60 and anti-SSB subphenotypes of pSS. **D** patients with ANA positivity had higher expression of TLR7 than those who were negative. *P < 0.05; ns, not signifcant. Anti-Ro52, anti-Ro/52 antibody; Anti-Ro60, anti-Ro/60 antibody; Anti-SSB, anti-SSB antibody; ANA, antinuclear antibodies

**Fig. 4:**
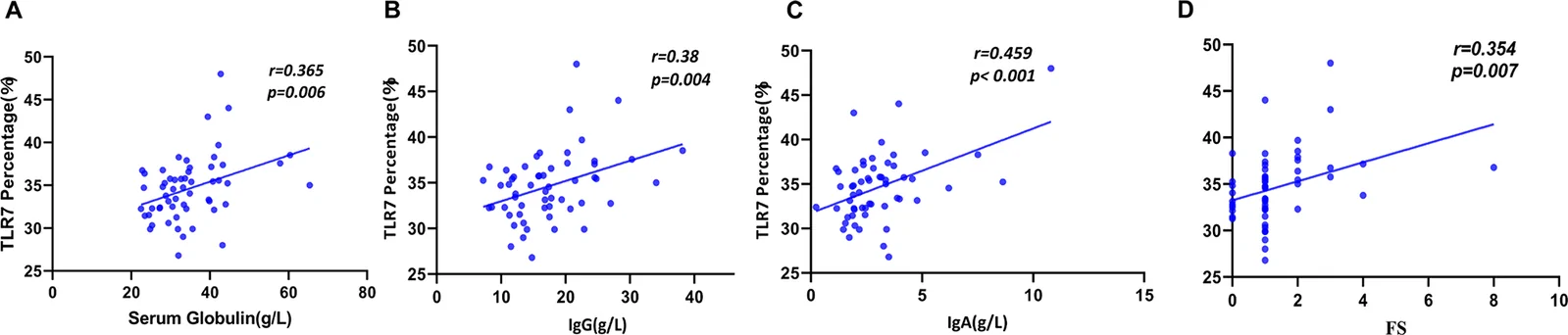
Correlations between TLR7 expression and IgG and serum globulin. **A** TLR7 expression was positively associated with IgG. **B** TLR7 expression was positively associated with serum globulin. **C** TLR7 expression was positively associated with IgA. **D** The staining intensity of TLR7 was positively correlated with the FS of pSS. IgG, immunoglobulin G; IgA, immunoglobulin A; IgM, immunoglobulin M; FS, focus score

The correlations between TLR7 levels and autoantibodies, including anti-Ro/52 and anti-Ro/60 and anti-SSB antibodies and ANA, in pSS patients were analysed. For the pSS subgroups, patients with anti-Ro/52 antibody positivity and ANA positivity had higher expression of TLR7 than those who were negative (35.24 ± 3.72% vs. 32.55 ± 3.38%, *P* = 0.0028; 35.03 ± 3.85% vs. 32.45 ± 2.76%, *P* = 0.046; Fig. 3A and D). There was higher expression of TLR7, while there was no statistically significant difference between the anti-Ro/60 and anti-SSB subtypes of pSS (*P* = 0.581 and *P* = 0.311; Fig. 3B and C). In addition, TLR7 levels in LSGs were positively correlated with serum globulin (*r* = 0.365, *p* = 0.006; Fig. 4A) and the levels of IgG (*r* = 0.38, *p* = 0.004; Fig. 4B) and IgA (*r* = 0.459, *p* < 0.001; Fig. 4C). These results support the positive correlation between TLR7 and autoantibody production in the development of pSS.

### TLR7 expression was correlated with glandular inflammatory activity in pSS

We further quantified the inflammatory infiltrate of LSGs by the focus score (FS) in pSS. The staining intensity of TLR7 was positively correlated with the FS in the LSGs of pSS patients (*r* = 0.354, *p* = 0.007; Fig. 4D). The results indicated that TLR7 expression was correlated with glandular inflammatory activity in pSS.

## Discussion

Salivary gland inflammation and focal lymphocyte infiltration have been identified as markers of pSS [16], although the inducing disease events and pathways that sustain chronic inflammation in salivary gland tissue remain unknown. It is hypothesized that the expression of danger signals caused by transient or persistent viral infection in epithelial cells leads to continued activation of TLR signals and ultimately to the pathogenesis of pSS [17]. The pathogenesis of pSS that has attracted much attention in recent years is the characteristic association with type I interferon (IFN). The type I IFN family consists of multiple members, including IFN-α and IFN-β, that are involved in a variety of biological functions, including defence against viral or bacterial infections, immune regulation, and negative regulation of proliferation. The activated type I IFN system called the interferon signature plays an important role in different autoimmune diseases, including pSS [12, 18].

As the most important endogenous nucleic acid sensitive TLR, TLR7 mediates transcription induction of type I IFN and other genes. If their activation is not controlled, it may lead to aseptic inflammation and autoimmunity [19]. In the current study, we examined TLR7 expression in LSGs from pSS patients. The semiquantitative analysis results showed that TLR7 was mainly expressed in ductal epithelial cells and infiltrative monocytes in LSGs and was more significantly increased in pSS patients. IL-6 levels may rise in the serum of systemic lupus erythematosus patients as the condition worsens [20]. Additionally, studies have shown that human Enterovirus 71 causes mice to develop neuropathy by causing IL-6 production by activating TLR7 signaling [21]. However, the efficacy of IL-6 receptor inhibitors in treating Sjögren’s syndrome is currently subpar, and further study is required to determine why [22].

Previous studies detected the expression of TLR7 and TLR9 in monocytes and the parotid gland in blood circulation and observed that they play a role in the characteristics of type I IFN, suggesting that induced expression of type I IFN inducers can enhance the response of TLR7 and TLR9 [23]. Animal experiments showed that NOD mice showed local salivary gland and lacrimal gland dysfunction and inflammation, lymph node and spleen enlargement, autoantibody positivity and other symptoms similar to Sjögren’s syndrome after the application of imiquimod, an agonist of TLR7 [24]. Anti-Ro/52, anti-Ro/60 and anti-La/SSB acted as endogenous ligands of TLR7 and activated B cells expressing TLR7 to differentiate into plasma cells. This resulted in the production of autoantibodies and immune complexes, demonstrating that TLR7 mediates pSS.

In this study, we showed that serum levels of IFN-α in pSS patients were significantly increased and correlated with TLR7 expression in LSGs. It is suggested that the pathogenic effect of TLR7 in salivary glands is related to innate immunity. It was also shown that the level of IL-6 in the serum of pSS patients was much higher, which was proven to be positively connected with TLR7 expression. Keratinocytes stimulated the production of inflammatory molecules, including IL-6, in the psoriasis mouse model via TLR2 and TLR7 signaling pathways, leading to illness [25]. This corresponds to our discovery trend. Surprisingly, we found that TLR7 was more markedly elevated in pSS and was correlated with increased autoantibody production, such as higher levels of serum globulin and increased IgG and IgA in serum, especially in the anti-Ro/52- and ANA-positive group.

We also found that the expression of TLR7 in LSGs increased as lymphocytic infiltration became more severe in pSS. This study attempted to reveal the correlation between TLR7 in LSGs and the clinical features of pSS. The findings support the previous argument that both systemic and local salivary gland autoimmune responses are causes of pSS salivary gland dysfunction. Systemic autoimmunity is highlighted by the detection of ANA, RF, anti-Ro/52, anti-Ro/60 and anti-La/SSB autoantibodies that are characteristic of primary pSS [26, 27].

This study has certain limitations. First, the sample size of the study is too small. Future studies in larger cohorts of patients with integrated analysis of different multiomics data would be valuable to support our study. Besides, transcriptional profiling [28] was performed and identified abnormal antigen presentation of dendritic cells and IFN signalling from pSS patients compared with Non-SS patients. However, this heterogeneous group of Non-SS patients shares objective dryness and occasionally presents single systemic features similar to patients with pSS. The control group we selected is similar to the previous study, and we cannot rule out that they may develop autoimmune diseases in the future. As such, longitudinal studies in the regulation of the immune profile of patients with Non-SS are essential to gain more insight into the mechanisms driving immune activation and development of pSS immunopathology.

## Conclusion

In summary, our study revealed that TLR7 may be involved in the local and systemic immune regulation of pSS, playing an important role in immune inflammation and the production of multiple antibodies. Further studies are needed to determine the specific mechanism of TLR7 in pSS.

## Data Availability

The data for the analyses in this study are available on reasonable request.
